# 

**Published:** 1900-03

**Authors:** 


					MARCH, 1900.
THE
AMERICAN JOURNAL
O F
DENTAL SCIENCE.
EDITED BY
F. J. S. GORGES,
RICHARD GRk335fiia6i?ae'i?Rflij
Vol. 33.
t h i mr"5~EnrrE*s
X?.-LL!
BALTIMORE!
SNOWDEN & COWMAN MFG. CO , 9 W. Fayette St.
LONDON:
TRUBNER &. CO., 60 Paternoster Row.
Entered at the Post Office at Baltimore, Md? as Second-class Matter.
PRYRBLE IN RDVPNCE.l
(PUBLISHED MONTHLY.I
$2.50 PER RNNUM.

				

